# Effects of dietary vitamin E on growth, immune response, antioxidant capacity, intestinal histomorphology, digestibility and disease resistance of juvenile Pacific white shrimp (*Penaeus vannamei*)

**DOI:** 10.1371/journal.pone.0333473

**Published:** 2025-09-30

**Authors:** Daehyun Ko, Nalin Medagoda, Jaebeom Shin, Mirasha Hasanthi, Jinho Bae, Byung Hwa Min, Kyeong-Jun Lee

**Affiliations:** 1 Department of Marine Life Sciences, Jeju National University, Jeju, South Korea; 2 Aquafeed Research Center, National Institute of Fisheries Science, Pohang, South Korea; 3 Marine Life Research Institute, Jeju National University, Jeju, South Korea; Tanta University Faculty of Agriculture, EGYPT

## Abstract

Vitamin E (tocopherols, VE) is a lipid-soluble antioxidant involved in neutralizing reactive oxygen species and maintaining immune function in animals. This study aimed to determine the optimum dietary VE requirement of juvenile Pacific white shrimp (*Penaeus vannamei*) for growth, feed utilization, immune responses, antioxidative capacity, diet digestibility, intestinal histomorphology and disease resistance against *Vibrio parahaemolyticus*. Eight experimental diets were formulated to contain graded levels of VE (0, 20, 40, 60, 80, 100, 120 and 240 mg/kg; designated as VE0, VE20, VE40, VE60, VE80, VE100, VE120 and VE240). Four replicate groups, each containing 30 shrimp (0.20 ± 0.04 g), were fed one of the diets six times daily for 56 days. Shrimp fed VE80 diet exhibited significantly increased growth performance compared to shrimp fed VE0, VE20 and VE240 diets. Non-specific immune responses were significantly enhanced in shrimp fed VE60-VE80 diets. Hepatopancreatic lipid peroxidation in VE80 group was significantly lower compared to the VE0 group. The expression of *Crustin*, *C-MnSOD* and *GPx* genes in the hepatopancreas was significantly upregulated in VE80 group. Graded dietary VE levels significantly linearly increased hemolymph and hepatopancreas VE concentrations. Intestinal villi height and width were significantly improved with dietary VE supplementation. The digestibility of protein, lipid and dry matter was significantly higher in shrimp fed VE80 diet compared to those fed VE0 diet. The resistance against *V. parahaemolyticus* was significantly higher in shrimp fed VE80, VE100 and VE120 diets compared to those fed VE0 and VE20 diets. The optimal dietary VE level for Pacific white shrimp was estimated to be 72.17 mg/kg for weight gain and 72.21 mg/kg specific growth rate, based on broken-line analysis. In conclusion, optimal dietary VE supplementation enhances shrimp growth, immunity, antioxidative defense and disease resistance against *V. parahaemolyticus*, thereby reducing the risk of early mortality syndrome caused by acute hepatopancreatic necrosis disease.

## Introduction

A well-balanced diet provides the nutrients necessary to support optimal growth, health, reproduction, stress resistance and disease tolerance in farm animals [1]. Micronutrients, such as vitamin E (VE), are vital for the biological and physiological functions of animals [2]. VE comprises a group of eight lipid-soluble compounds, known as tocopherols and tocotrienols, with α-tocopherol being the most biologically active and abundant form [3]. Consequently, α-tocopherol is significantly accumulated in the body and is abundant in animal tissues [3]. Adequate dietary intake of VE is crucial as animals cannot synthesize it *in vivo* [4].

VE is well-known for its antioxidant properties and it is primarily located in the phospholipid bilayer of cell membranes, where it binds to fatty acids owing to its lipid-soluble nature [5]. Polyunsaturated fatty acids are highly susceptible to oxidation, leading to the formation of reactive aldehydes such as malondialdehyde (MDA) and 4-hydroxynonenal [6]. VE donates electrons to neutralize lipid peroxyl radicals in the lipid phase. Furthermore, VE and vitamin C interact synergistically, with each molecule recovering the other by exchanging hydrogen atoms during their neutralization of free radicals [2]. Beyond its antioxidative properties, VE is essential for regulating several biological functions including smooth muscle cell proliferation and protein kinase C activity [2]. Dietary VE supplementation improved non-specific immune responses, antioxidant and anti-inflammatory-related gene expression in grass carp (*Ctenopharyngodon idella*) enhancing their resistance to *Aeromonas hydrophila* [7].

Dandapat et al. [8] demonstrated that dietary supplementation of VE significantly improved the antioxidant capacity of giant freshwater prawns (*Macrobrachium rosenbergii*). Similarly, Lee and Shiau [9] reported increased growth, hematocyte count and superoxide dismutase (SOD) activity along with reduced lipid peroxidation in black tiger shrimp (*Penaeus monodon*) following VE administration. In Pacific white shrimp (*Penaeus vannamei*), dietary VE supplementation significantly enhanced antioxidative enzyme and Na^+^/K^+^-ATPase activities [10]. However, a decreasing trend in antioxidative enzyme activity and MDA levels was observed in the hepatopancreas of Oriental river prawn (*Macrobrachium nipponense*) with increasing dietary VE levels [11]. Wang et al. [12] found that the dietary inclusion of astaxanthin and VE significantly improved the growth and pigmentation of Kuruma shrimp (*Marsupenaeus japonicus*).

Pacific white shrimp accounts for 53% of global farmed crustacean production, with an annual production of 6.8 million tons valued at approximately 26.7 billion USD in 2022 [13]. It is the most widely cultured shrimp species due to its rapid growth, high survival and strong environmental tolerance. The assessment of optimal dietary nutrients levels for most cultured species is essential to sustainable aquaculture under intensive culture conditions. To our knowledge, no previous study has comprehensively evaluated the effect of dietary VE on feed utilization, immunity, gut morphology, diet digestibility, tissue VE deposition and disease resistance to *Vibrio parahaemolyticus*, as earlier studies primarily focused on growth and antioxidant activity [14]. Therefore, this study aimed to determine the optimal dietary VE (DL-α-tocopherol acetate) requirement of juvenile Pacific white shrimp for growth performance, feed utilization, antioxidant capacity, innate immunity, gut histomorphology, diet digestibility and tissue compositions of Pacific white shrimp. In particular, we assessed the effect of dietary VE supplementation on resistance to *V. parahaemolyticus* infection, with the potential to reduce the risk of early mortality syndrome (EMS) caused by acute hepatopancreatic necrosis disease (AHPND).

## Materials and methods

### Ethics statement

The protocols and animal handling procedures used for this feeding trial, digestibility study and challenge test were designed following national and international animal handling guidelines and protocols were evaluated and approved by the Animal Care and Use Committee of Jeju National University (IACUC approval number – 2024−0083).

### Experimental diets

A basal diet designated as VE0 (crude protein, 34.8%; crude lipid, 8.7%; [15]) was formulated based on the practical ingredients, including fish meal (sardine), tuna byproduct meal, soybean meal and squid liver powder as protein sources, with cod liver oil serving as the lipid source (Table 1). Wheat flour and starch were used as the carbohydrate source and binding agents. Lecithin, vitamin premixture (excluding VE), mineral premixture, cholesterol and monocalcium phosphate were incorporated as additional additives. VE0 diet was supplemented with seven different levels of VE (DL-α-tocopherol acetate, DSM, Netherlands) by 20, 40, 60, 80, 100, 120 and 240 mg/kg while concomitantly reducing cellulose level to prepare VE20, VE40, VE60, VE80, VE100, VE120 and VE240 diets. The wet dough was prepared by mixing dry ingredients, the relevant amount of cod liver oil and 15% distilled water. Then, the dough was pelletized (2 mm; SP-50, Kumkang ENG, Daegu, South Korea) and air-dried at 23 °C in a feed dryer (SI-2400, Shinil General Dryer, Daegu, South Korea) for 8 h.

**Table 1:** Formulation and chemical composition of the experimental diets for juvenile Pacific white shrimp (*Penaeus vannamei*) (g/kg, dry matter basis).

Ingredients	Experimental diets							
	VE0	VE20	VE40	VE60	VE80	VE100	VE120	VE240
Fish meal (sardine)^1^	50	50	50	50	50	50	50	50
Tuna byproduct meal^2^	50	50	50	50	50	50	50	50
Squid liver powder	50	50	50	50	50	50	50	50
Soybean meal	400	400	400	400	400	400	400	400
Vitamin E (50%)^3^	0	0.04	0.08	0.12	0.16	0.20	0.24	0.48
Cellulose	8	7.96	7.92	7.88	7.84	7.80	7.76	7.52
Starch	140	140	140	140	140	140	140	140
Wheat flour^4^	180	180	180	180	180	180	180	180
Cod liver oil^5^	50	50	50	50	50	50	50	50
Lecithin	10	10	10	10	10	10	10	10
Vitamin premix (excluding vitamin E)^6^	10	10	10	10	10	10	10	10
Mineral premix^7^	20	20	20	20	20	20	20	20
Cholesterol	2	2	2	2	2	2	2	2
Monocalcium phosphate	30	30	30	30	30	30	30	30
Analyzed chemical composition (%)								
Crude protein	34.8	34.7	35.0	34.9	35.0	34.8	34.8	35.0
Crude lipid	8.7	8.6	8.6	8.7	8.6	8.6	8.5	8.6
Ash	9.1	9.2	9.6	9.1	9.3	9.7	9.3	9.1
Dry matter	92.6	92.6	92.5	92.8	92.6	92.7	92.5	92.6
Vitamin E level (mg/kg)	14.4	26.2	47.4	59.9	82.9	99.3	134.8	260.4

1, 2 Orizon S.A., Corp., Santiago, Chile (crude protein: 69, 55%). ^3^DL-α-Tocopherol acetate, DSM, Netherlands. ^4^Daehan Flour, Incheon, South Korea. ^5^E-wha Oil & Fat Industry Corp., Busan, South Korea. ^6^Vitamin premix contains (g/kg): β-carotene, 0.57; cholecalciferol, 0.0004; ascorbic acid, 10.0; menadione, 0.9; thiamine hydrochloride, 8.9; riboflavin, 6.9; pyridoxine, 7.2; cyanocobalamin, 0.09; inositol, 45.0; pantothenic acid, 18.0; biotin, 0.9; niacinamide, 18.0; folic acid, 7.2. ^7^Mineral premix contains (g/kg): ferrous sulfate, 10; copper sulfate, 1; zinc sulfate, 3; manganese sulfate, 2; cobalt chloride, 10; potassium iodide, 1; potassium, 6; sodium selenite, 0.01.

Following the method described by Tangney et al. [16], the VE concentrations in triplicate diet samples were analyzed using high-performance liquid chromatography (HPLC). A 2 g sample was homogenized in 5 mL of ethanol for 10 min. Then, 2.5 mL of n-hexane was added, thoroughly mixed for 1 min and, centrifuged at 3000 × *g* for 5 min. The supernatant was passed through a 0.2 µm membrane filter and isopropanol was added subsequently. A separation module (model e2695, Waters, USA) coupled with a photodiode array detector (model 2998, Waters, USA) was used for HPLC analysis. An analytical column (C18, 5 µm, 4.6 mm × 250 mm, Waters-SYMMETRY^®^, USA) was used for compound separation and the mobile phase was 95% methanol. The excitation wavelength was set at 298 nm and the detection wavelength at 325 nm. The VE concentrations in the diets were determined as 14.4, 26.2, 47.4, 59.9, 82.9, 99.3, 134.8 and 260.4 mg/kg, respectively. The moisture and ash levels of the diet were measured following the methods published by AOAC [17]. Crude protein level was assessed using a Kjeltec Analyzer Unit 2300 (nitrogen% × 6.25, FOSS, Sweden), while crude lipid was assessed by SOX406 (Jinan Hanon Instruments, Shandong, China).

### Experimental design and feeding trial

Shrimp were obtained from a private shrimp hatchery (Jeju, South Korea) and acclimated to the experimental conditions in the Marine Life Research Institute (Jeju, South Korea) for two weeks prior to the start of the feeding trial. During the acclimation period, a commercial feed (crude protein 40%, crude lipid 9%; CJ Cheiljedang, Seoul, South Korea) was provided. A batch of similar sized shrimp was selected for the feeding trial. Thirty-two tanks were arranged in a completely randomized design to form quadruplicate groups per treatment, and a set of 30 shrimp (initial mean body weight: 0.20 ± 0.04 g) was placed into each tank (180 L). Experimental diets were randomly allocated to tanks and fed for 56 days. The biomass in each tank was measured at two-week intervals to adjust the feed ration. Shrimp were fed 5–12% of tank biomass based on careful observation of their feed intake for the subsequent two weeks. The daily feed amount was divided into six equal portions and fed at 08:30, 10:30, 12:30, 14:30, 16:30 and 18:30 h. Following feeding, uneaten feed pellets were collected by siphoning 20–25 min after feeding, and their dry weight was determined to calculate the exact feed intake. The culture tanks were continuously aerated to maintain a dissolved oxygen level of 6.65 ± 0.44 mg/L and water temperature was maintained at 30 ± 1 °C using automatic aquarium heaters. To maintain water quality, 70% of rearing water was replaced twice a week with pre-heated and filtered seawater. Water quality parameters were maintained: pH, 7.07 ± 0.06; salinity, 31.5 ± 0.3 ppt and ammonia concentration, 0.039 ± 0.004 mg/L, respectively.

### Sample collection and analysis

At the end of the feeding trial, the final body weight (FBW) of individual shrimp in each tank was measured to evaluate growth performance and feed utilization. Subsequently, six shrimp per tank were selected and euthanized by immersion in ice-cold water. Hemolymph was extracted from the ventral sinus of each shrimp using a 1 mL syringe (24 gauge), then immediately combined with a double volume of anticoagulant solution (A3551, Alsever’s solution, Sigma-Aldrich, USA) and stored at −83 °C for further analyses. Six hepatopancreas samples from each tank were collected under sterile conditions and stored at −83 °C for subsequent analysis of gene expression, tissue VE and MDA levels. Three intestine samples, each measuring 1 cm in length, were obtained from the shrimp used for hepatopancreas collection, 0.5 cm posterior to the hepatopancreas and preserved in Davidson solution for histological analysis.

Immune parameters were analyzed using hemolymph plasma samples. Nitro-blue tetrazolium (NBT), lysozyme, phenoloxidase (PO) and antiprotease activities were assessed following Zhang et al. [18], Hultmark [19], Hernández-López et al. [20] and Ellis [21] methods, respectively. MDA level in the hepatopancreas was determined using commercial thiobarbituric acid reactive substances (TBARS) assay kits (Biovision, Milpitas, USA).

Those procedures are briefly explained below. The procedure for assessing NBT activity involved the addition of 50 µL of hemolymph to 200 µL of Hank’s Balanced Salt solution, followed by incubation at 25 °C for 30 min. Subsequently, 100 µL of Zymosan (0.1% in Hank’s solution) was introduced, and the mixture was incubated at 37 °C for 2 h. Thereafter, 100 µL of NBT solution (0.3%) was added, and the sample was incubated at 37 °C for an additional 2 h. Following this, 600 µL of methanol (100%) was added, and the mixture was centrifuged, with the supernatant being discarded. The pellet was washed three times with 100 µL of methanol (70%) and allowed to dry for 5 min. Subsequently, 700 µL of KOH (2M) and 800 µL of dimethyl sulfoxide were added, and the absorbance was measured at 620 nm.

The lysozyme assay involved the combination of 20 µL of plasma with 200 µL of *Micrococcus lysodeikticus* (0.75 mg/mL in phosphate buffer, pH 6.4) in a 96-well plate. The samples were incubated at 37 °C for 0 and 60 min, and the reduction in absorbance was measured at 570 nm.

To assess PO activity, 50 µL of plasma was combined with 50 µL of trypsin (0.1 mg/mL in cacodylate buffer, pH 7) in a 96-well plate and incubated at 25 °C for 10 min. Subsequently, L-DOPA (3 mg/mL in cacodylate buffer, pH 7) was introduced, and the mixture was incubated at 25 °C for an additional 10 min. The absorbance was measured at 492 nm.

Antiprotease activity was analyzed using the following procedure. A volume of 20 µL of plasma was combined with 20 µL of trypsin (50 mM, 5 mg/mL in Tris-HCl) and incubated at 24 °C for 10 min. Subsequently, 200 µL of phosphate buffer (0.1 M) and 250 µL of Azocasein (20 mg/mL in NaOH) were added, followed by incubation at 24 °C for 1 h. The mixture was then treated with 500 µL of trichloroacetic acid solution (100 mg/mL in ultra-pure water) and incubated at 24 °C for 30 min. The sample underwent centrifugation (6,000 × g, 5 min, 4 °C), after which 100 µL of the supernatant was mixed with 100 µL of NaOH (40 mg/mL in ultra-pure water). The absorbance was measured at 450 nm.

MDA levels of hepatopancreas tissues were analyzed following the manufacturer’s guidelines provided with above mentioned commercial kit. Hepatopancreas tissue sample (approximately 10–20 mg) was homogenized on ice in 300 µL of phosphate-buffered saline containing 1 × butylated hydroxytoluene. The homogenate was centrifuged at 13,000 × g for 10 min at 4 °C to remove insoluble material. A total of 200 µL of the resulting supernatant was transferred into a microcentrifuge tube. For the assay, 600 µL of thiobarbituric acid reagent was added to each vial containing either the sample or MDA standards. The mixtures were incubated at 95 °C for 60 min and then cooled in an ice bath for 10 min. A volume of 200 µL from each reaction mixture (total 800 µL) was transferred to a 96-well microplate, and the absorbance was measured at 532 nm using a microplate reader.

### Reverse transcription real-time quantitative polymerase chain reaction (RT-qPCR) for relative gene expression analysis

Total RNA was extracted by homogenizing approximately 80–100 mg of hepatopancreas samples (*n* = 6) with a hand-held homogenizer in TRIzol reagent (T9424, Sigma-Aldrich, USA). RNA purity and concentration were assessed using a μDrop^TM^ plate spectrophotometer (Thermo Fisher Scientific, USA). PrimeScript First Strand cDNA Synthesis Kit (TaKaRa, Shiga, Japan) was employed to synthesize complementary DNA. Primer sequences for *β-actin* (housekeeping gene), *Crustin*, cytosolic-manganese SOD (*C-MnSOD*) and glutathione peroxidase (*GPx*) were designed based on Pacific white shrimp DNA sequences published in the NCBI GenBank^®^ genetic sequence database (Table 2). The RT-qPCR protocol was initiated with a denaturation step at 95 °C for 10 min, followed by 40 amplification cycles consisting of 5 s at 95 °C, 10 s at 58 °C and 20 s at 72 °C. Relative mRNA expression levels were determined following the 2^−ΔΔCT^ method, referring to the VE0 group.

**Table 2:** Primers used for gene expression analysis.

Gene name	Oligonucleotide sequence	GenBank^®^ identifier
*Crustin*-F	GAGGGTCAAGCCTACTGCTG	AY488497
*Crustin*-R	ACTTATCFAFFCCAFCACAC	
*C-MnSOD*-F	TCATGCTTTGCCACCTCTC	DQ029053
*C-MnSOD*-R	CCGCTTCAACCAACTTCTTC	
*GPx*-F	AGGGACTTCCACCAGATG	AY973252.2
*GPx*-R	CAACAACTCCCCTTCGGTA	
*β-actin*-F	GAGCAACACGGAGTTCGTTGT	AF300705.2
*β-actin*-R	CATCACCAACTGGGACGACATGGA	

*C-MnSOD*, cytosolic manganese superoxide dismutase; *GPx*, glutathione peroxidase.

### Histological analysis

Histological analysis was conducted according to the protocol established by Fischer et al. [22]. Intestine samples were transferred from Davidson solution to 70% ethanol within 24 h after sampling and dehydrated in a gradient ethanol series. Dehydrated samples were embedded in paraffin blocks using an embedding station (Leica EG1150, Leica Biosystems, USA) and sectioned into 5 μm thickness using a rotary microtome (Leica RM2235, Leica Biosystems, USA). Tissue sections were stained with hematoxylin and eosin and examined under a light microscope at 40× magnification (Leica DM750. Leica Microsystems, South Korea). Villus height, villus width and submucosa thickness were measured using image analysis software (Leica Application Suite, Version 4.13.0, Switzerland).

### Digestibility test

Diets for the digestibility test were prepared by incorporating 1% chromium oxide (Cr_2_O_3,_ Sigma-Aldrich, USA) as an inert indicator, following the above diet preparation process. A total of 480 shrimp, with an average weight of 4.62 ± 0.45 g, were stocked into 32 tanks, each with a capacity of 120 L, in quadruplicates. The shrimp were acclimated to the experimental conditions for five days by feeding them with the respective experimental diet. Shrimp were fed at 5% of body weight during the feces collection period, with feeding times at 09:00, 13:00 and 17:00 h. Uneaten feed and fecal residues were removed 30 min after each feeding. Each morning at 08:30 h, 70% of the water in tanks was exchanged, removing any possible debris on the bottom of each tank. Fecal samples were then collected at 10:30, 14:30 and 18:30 h for two weeks using a glass Pasteur pipette. The fecal matter samples were filtered using Whatman filter paper and stored in a freezer at −83 °C daily until the feces collection was terminated. Collected fecal matter samples from each tank were pooled and subsequently freeze-dried. Apparent digestibility coefficients (ADCs) of protein (ADCp), lipid (ADCl) and dry matter (ADCd) were determined by the method described in Divakaran et al. [23]. The ADCs were calculated using the following formulas:

ADCd = 100 – (% Cr_2_O_3_ in diet/ % Cr_2_O_3_ in feces) ×100

ADCs of nutrients = ADCd × (% nutrient in feces/ % nutrient in diet)

### Challenge test

*V. parahaemolyticus* strain 13–028/A3, obtained from the Korean Culture Collection of Aquatic Microorganisms (Busan, South Korea), was used as the pathogen for the challenge test. The strain was confirmed as *VP*_AHPND_ through a duplex PCR assay targeting the *pir*A- and *pir*B-like genes following Ko et al. [24]. The bacterium was cultured in tryptic soy broth (TSB) supplemented with NaCl and incubated at 30 °C for 18 h with agitation at 150 rpm. A preliminary test was conducted to determine the LD_50_ inoculation level of *V. parahaemolyticus* suspension. For the challenge test, 45 shrimp were randomly selected from each treatment group and distributed randomly into 3 tanks (120 L) per diet with 15 shrimp per tank in a quarantine room. Then, each tank was inoculated with 50 mL of *V. parahaemolyticus* suspension (2.8 × 10^6^ CFU/mL). Three additional tanks were prepared as negative controls containing the same number of randomly selected shrimp and inoculated with 50 mL of pure TSB medium. Shrimp mortality was monitored hourly for the first 48 h, and then every 4 h thereafter until the termination of the test (240 h). After 24 h, 70% of the rearing water in each tank was exchanged. During the challenge test period, shrimp were fed their respective diet at 5% of body weight at 08:30, 13:30 and 18:30 h.

### Statistical analysis

Data were presented as means ± SD and percentage data were arcsine-transformed prior to statistical analysis. The data for each parameter were initially checked for normality using the Shapiro-Wilk test. Statistical comparisons were conducted using one-way ANOVA with SPSS version 21.0 (SPSS Inc., Chicago, IL, USA) to detect significant differences among treatments. Post-hoc comparisons of significant means identified by ANOVA were performed using Duncan’s multiple range test, with a significance level set at **P* *< 0.05. Broken-line regression analysis was employed to determine the optimum VE levels for weight gain (WG, %) and specific growth rate (SGR, %) [25]. Orthogonal polynomial contrasts were applied to assess whether the effects of VE on each parameter followed the linear or quadratic trend.

## Results

### Growth performance, feed utilization and survival

**Fig 1:**
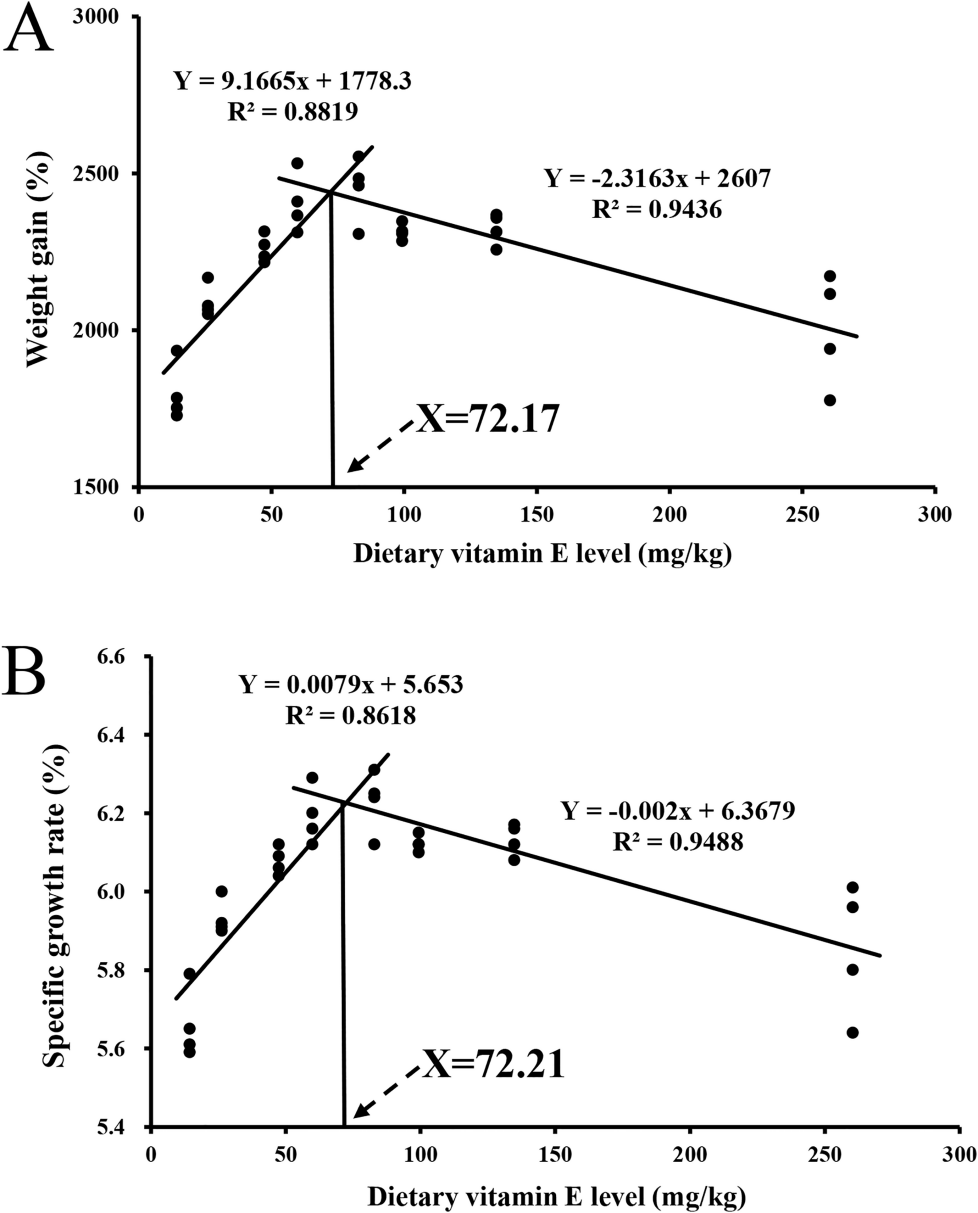
Broken-line analysis of the relationship between dietary vitamin E level and growth performance of juvenile Pacific white shrimp (*Penaeus vannamei*). (A) The regression analysis between weight gain (%) and dietary vitamin E level; (B) The regression analysis between specific growth rate (%) and dietary vitamin E level. Data represent the means of four replicates in each treatment (n = 4): X represents the optimum dietary vitamin E requirement.

Shrimp in all groups accepted diets readily. FBW was significantly higher in VE80 group than in VE0, VE20, VE40 and VE240 groups (Table 3). WG and SGR were significantly improved in VE60, VE80 and VE120 groups than in VE0, VE20 and VE240 groups. FBW, WG and SGR showed significant linear and quadratic trends in response to the increasing dietary VE levels. Shrimp fed VE100 diet demonstrated significantly higher survival than those fed VE0 diet, with survival exhibiting a significant linear trend. However, dietary VE did not significantly affect feed conversion ratio (FCR) and protein efficiency ratio (PER). According to broken-line regression analysis of WG and SGR, the optimal dietary VE requirements for the shrimp were determined to be 72.17 mg/kg and 72.21 mg/kg, respectively (Fig 1).

**Table 3:** Growth performance, feed utilization and survival of juvenile Pacific white shrimp (*Penaeus vannamei*) fed different vitamin E levels incorporated into the diets for 56 days.

Diets	FBW^1^	WG^2^	SGR^3^	FCR^4^	PER^5^	Survival (%)
VE0	3.80 ± 0.18^e^	1800 ± 93^d^	5.66 ± 0.09^d^	1.43 ± 0.06	2.01 ± 0.09	92.5 ± 3.2^b^
VE20	4.39 ± 0.10^cd^	2091 ± 53^bc^	5.94 ± 0.05^bc^	1.37 ± 0.02	2.09 ± 0.03	96.7 ± 3.9^ab^
VE40	4.70 ± 0.09^bc^	2260 ± 44^ab^	6.08 ± 0.04^ab^	1.36 ± 0.02	2.11 ± 0.03	97.5 ± 3.2^ab^
VE60	5.03 ± 0.17^ab^	2405 ± 94^a^	6.19 ± 0.07^a^	1.32 ± 0.04	2.18 ± 0.06	96.7 ± 4.7^ab^
VE80	5.13 ± 0.19^a^	2451 ± 104^a^	6.23 ± 0.08^a^	1.34 ± 0.07	2.12 ± 0.11	95.6 ± 1.9^ab^
VE100	4.83 ± 0.06^ab^	2314 ± 26^a^	6.12 ± 0.02^ab^	1.35 ± 0.02	2.13 ± 0.03	100 ± 0.0^a^
VE120	4.85 ± 0.09^ab^	2324 ± 51^a^	6.13 ± 0.04^a^	1.38 ± 0.01	2.08 ± 0.02	98.3 ± 3.3^ab^
VE240	4.21 ± 0.36^de^	2001 ± 179^cd^	5.85 ± 0.17^c^	1.45 ± 0.13	2.07 ± 0.18	99.2 ± 1.7^ab^
Pr > F^*^						
ANOVA	<0.001	<0.001	<0.001	0.067	0.288	0.048
Linear	<0.001	<0.001	<0.001	0.699	0.560	0.015
Quadratic	<0.001	<0.001	<0.001	0.001	0.014	0.386

Initial mean body weight of shrimp was 0.20 ± 0.04 g. The experimental diets were formulated to contain vitamin E at 0, 20, 40, 60, 80, 100, 120 and 240 mg/kg (designated as VE0, VE20, VE40, VE60, VE80, VE100, VE120 and VE240, respectively).

Values are the means of quadruplicate groups and are presented as mean ± SD. Values with different superscript letters in the same column are significantly different (**P* *< 0.05).

*Significance probability associated with the *F*-statistic.

^1^Final body weight (g). ^2^Weight gain (%) = [(final body weight – initial body weight)/ initial body weight] × 100. ^3^Specific growth rate (%) = [(ln(final body weight) – ln(initial body weight))/ days] × 100. ^4^Feed conversion ratio = dry feed intake (g)/ wet weight gain (g). ^5^Protein efficiency ratio = wet weight gain (g)/ total protein intake (g).

### Non-specific immune responses

NBT activity was significantly elevated in shrimp fed VE60 diet compared to those fed VE0 diet (Table 4). MDA level was significantly lower in VE80 group than in VE0, VE120 and VE240 groups. Shrimp fed VE20, VE40, VE60 and VE80 diets showed significantly improved lysozyme activity than those fed VE0 diet. PO activity was significantly increased in VE60 group compared to VE0, VE20, VE80, VE100, VE120 and VE240 groups. Antiprotease activity was significantly increased in VE80 group compared to all other groups. MDA level demonstrated significant linear and quadratic trends. NBT, lysozyme, PO and antiprotease activities exhibited a significant quadratic trend in response to dietary VE levels.

**Table 4:** Non-specific immune responses and lipid peroxidation status of juvenile Pacific white shrimp (*Penaeus vannamei*) fed different vitamin E levels incorporated into the diets for 56 days.

Diets	NBT^1^	Antiprotease^2^	Lysozyme^3^	PO^4^	MDA^5^
VE0	2.41 ± 0.16^b^	4.52 ± 2.30^c^	3.55 ± 0.29^b^	0.106 ± 0.023^c^	2.41 ± 0.11^b^
VE20	2.62 ± 0.15^ab^	9.66 ± 2.33^b^	4.85 ± 0.34^a^	0.139 ± 0.020^bc^	2.31 ± 0.68^bc^
VE40	2.84 ± 0.11^ab^	7.14 ± 2.55^bc^	4.82 ± 0.57^a^	0.155 ± 0.028^ab^	1.48 ± 0.29^bc^
VE60	3.29 ± 0.07^a^	9.38 ± 1.51^b^	4.89 ± 0.52^a^	0.182 ± 0.030^a^	1.22 ± 0.11^bc^
VE80	2.69 ± 0.07^ab^	14.4 ± 2.95^a^	4.97 ± 0.33^a^	0.122 ± 0.024^bc^	1.11 ± 0.06^c^
VE100	2.81 ± 0.36^ab^	6.73 ± 2.52^bc^	4.13 ± .058^ab^	0.133 ± 0.022^bc^	1.89 ± 1.13^bc^
VE120	2.80 ± 0.44^ab^	8.78 ± 1.00^b^	4.18 ± 0.32^ab^	0.119 ± 0.016^bc^	3.90 ± 0.06^a^
VE240	2.61 ± 0.15^ab^	7.80 ± 1.86^bc^	4.23 ± 0.79^ab^	0.118 ± 0.026^bc^	3.90 ± 0.81^a^
Pr >F^*^					
ANOVA	0.045	<0.001	0.014	0.020	<0.001
Linear	0.307	0.120	0.703	0.418	0.001
Quadratic	0.007	<0.001	0.001	0.005	<0.001

The experimental diets were formulated to contain vitamin E at 0, 20, 40, 60, 80, 100, 120 and 240 mg/kg (designated as VE0, VE20, VE40, VE60, VE80, VE100, VE120 and VE240, respectively).

Values are the means of quadruplicate groups and are presented as mean ± SD. Values with different superscript letters in the same column are significantly different (**P* *< 0.05).

*Significance probability associated with the *F*-statistic.

^1^Nitroblue tetrazolium activity (absorbance at 540 nm). ^2^Antiprotease activity (%, inhibition).

^3^Lysozyme activity (µg/mL). ^4^Phenoloxidase activity (absorbance at 492 nm). ^5^Malondialdehyde level in the hepatopancreas (nmol/mg protein).

### Expression of immune and antioxidant-related genes

**Fig 2:**
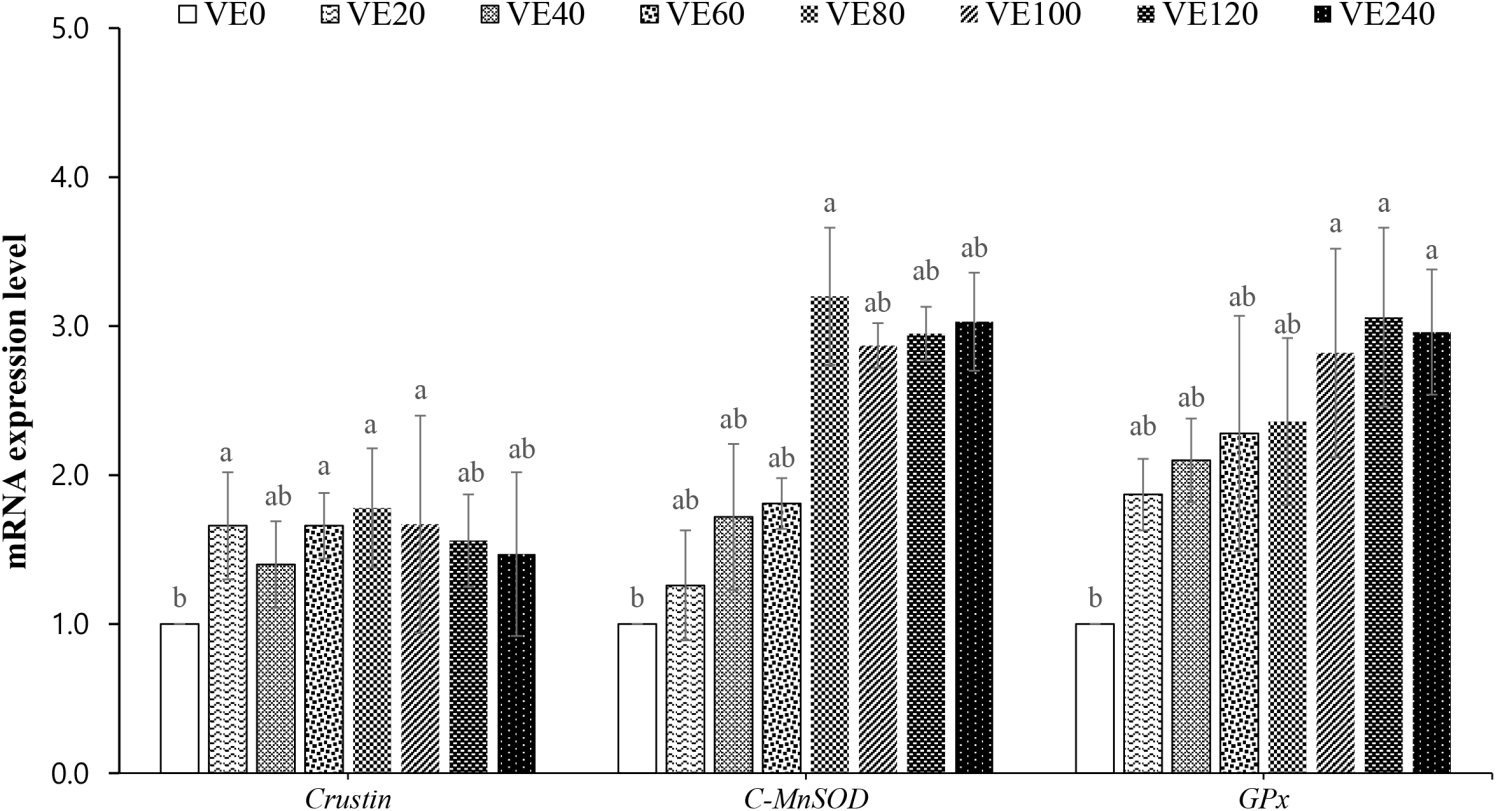
Immune and antioxidant-related gene expressions of *Crustin*, cytosolic-manganese superoxide dismutase (*C-MnSOD*) and glutathione peroxidase (*GPx*) of juvenile Pacific white shrimp (*Penaeus vannamei*) fed graded levels of vitamin E for 56 days. The experimental diets were formulated to contain vitamin E at 0, 20, 40, 60, 80, 100, 120 and 240 mg/kg (designated as VE0, VE20, VE40, VE60, VE80, VE100, VE120 and VE240, respectively). Bars with different letters indicate significant differences (*P *< 0.05).

The immune and antioxidant-related gene expressions of the shrimp fed the experimental diets are presented in Fig 2. The relative gene expression of *Crustin* was significantly higher in VE20, VE60, VE80 and VE100 groups compared to VE0 group. *C-MnSOD* gene expression was significantly upregulated in VE80 group than in VE0 group. Relative expression of *GPx* gene was significantly higher in VE100, VE120 and VE240 groups compared to VE0 group.

### VE concentration in hemolymph and hepatopancreas

Hemolymph and hepatopancreas VE levels were significantly increased with increasing levels of VE in the diet (Table 5). VE240 group showed significantly increased hemolymph VE levels than VE0, VE20, VE40, VE60, VE80 and VE100 groups. VE240 group demonstrated significantly higher hepatopancreas VE levels compared to all other groups. VE40, VE60, VE80 and VE100 groups exhibited no significant differences in hepatopancreas VE levels among the groups; however, VE levels in these groups were significantly lower than those in VE120 group. Hemolymph VE levels had a significant linear trend, whereas the hepatopancreas VE levels exhibited both significant linear and quadratic trends to dietary VE levels.

**Table 5:** Tissue vitamin E concentrations (μg/g) of juvenile Pacific white shrimp (*Penaeus vannamei*) fed different vitamin E levels incorporated into the diets for 56 days.

Diets	Vitamin E concentrations	
	Hemolymph	Hepatopancreas
VE0	0.35 ± 0.24^d^	5.05 ± 0.1^e^
VE20	0.77 ± 0.32^cd^	71.4 ± 21^ed^
VE40	1.02 ± 0.29^cd^	168 ± 28^cd^
VE60	1.27 ± 0.17^bcd^	177 ± 3^cd^
VE80	1.80 ± 0.64^bc^	227 ± 53^cd^
VE100	2.08 ± 0.25^bc^	204 ± 34^c^
VE120	2.45 ± 0.16^ab^	370 ± 33^b^
VE240	3.43 ± 0.40^a^	623 ± 53^a^
Pr >F^*^		
ANOVA	<0.001	0.001
Linear	<0.001	<0.001
Quadratic	0.143	0.003

The experimental diets were formulated to contain vitamin E at 0, 20, 40, 60, 80, 100, 120 and 240 mg/kg (designated as VE0, VE20, VE40, VE60, VE80, VE100, VE120 and VE240, respectively).

Values are the means of quadruplicate groups and are presented as mean ± SD. Values with different superscript letters in the same column are significantly different (**P* *< 0.05).

*Significance probability associated with the *F*-statistic.

### Intestinal histology and digestibility

**Fig 3:**
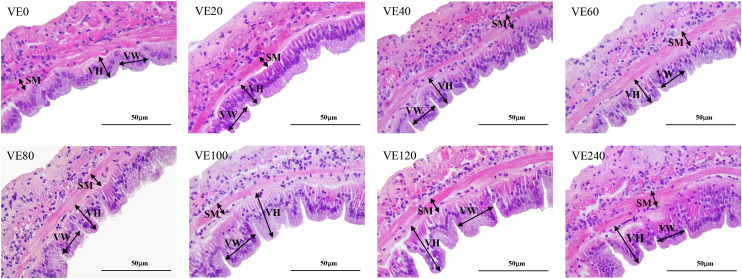
The intestinal histomorphology of juvenile Pacific white shrimp (*Penaeus vannamei*) fed different levels of vitamin E (DL-α-tocopherol acetate) incorporated into the diet for 56 days. Representative histological images of hematoxylin and eosin-stained sections of intestines were obtained under 40× magnification of the light microscope. The experimental diets were formulated to contain vitamin E at 0, 20, 40, 60, 80, 100, 120 and 240 mg/kg (designated as VE0, VE20, VE40, VE60, VE80, VE100, VE120 and VE240, respectively). villi height (VH); villi width (VW); submucosa thickness (SM).

The results of the intestinal histology are presented in Table 6 and Fig 3. Villi height was significantly increased in shrimp fed VE120 diet than in shrimp fed VE0, VE20 and VE40 diets. VE40, VE60, VE80, VE100, VE120 and VE240 groups showed significantly increased villi width compared to VE0 and VE20 groups. However, dietary VE did not significantly affect submucosa thickness. Both linear and quadratic trends in villi height and width were significant with the increase in dietary VE levels. ADCd was significantly higher in VE80 group than in VE0, VE120 and VE240 groups (Table 6). Similarly, ADCp was significantly higher in VE80 diet compared to VE0, VE100, VE120 and VE240 groups. Shrimp fed VE20 and VE80 diets showed significantly improved ADCl than shrimp fed VE0 and VE100 diets. ADCd showed a significant quadratic trend, whereas ADCp exhibited a significant linear trend. ADCl showed a significant linear and quadratic trend.

**Table 6:** Intestinal morphology and nutrient digestibility of the diets of juvenile Pacific white shrimp (*Penaeus vannamei*).

Diets	Intestinal morphology			Apparent digestibility coefficients (%)		
	Villi height (µm)	Villus width (µm)	Submucosa thickness (µm)	ADCd^1^	ADCp^2^	ADCl^3^
VE0	23.7 ± 2.2^c^	22.4 ± 1.1^b^	8.43 ± 0.70	71.9 ± 2.4^c^	85.1 ± 0.8^b^	90.2 ± 2.4^b^
VE20	22.8 ± 2.4^c^	22.9 ± 0.6^b^	8.97 ± 0.68	78.2 ± 2.2^abc^	88.1 ± 0.9^ab^	94.3 ± 1.8^a^
VE40	27.3 ± 0.8^bc^	25.5 ± 0.9^a^	8.23 ± 0.81	78.7 ± 2.3^ab^	88.2 ± 2.4^ab^	92.9 ± 1.3^ab^
VE60	27.4 ± 2.3^ab^	25.6 ± 0.4^a^	9.33 ± 0.85	79.1 ± 3.8^ab^	87.8 ± 2.7^ab^	93.2 ± 0.6^ab^
VE80	29.4 ± 1.4^ab^	26.1 ± 1.3^a^	9.47 ± 0.75	82.7 ± 1.9^a^	90.9 ± 0.5^a^	94.8 ± 1.3^a^
VE100	29.4 ± 1.3^ab^	25.7 ± 0.6^a^	9.37 ± 1.10	78.5 ± 2.4^ab^	86.4 ± 4.4^b^	91.4 ± 1.7^b^
VE120	30.2 ± 2.8^a^	26.2 ± 1.4^a^	9.27 ± 0.85	75.2 ± 3.1^bc^	86.0 ± 0.8^b^	92.7 ± 0.7^ab^
VE240	29.3 ± 2.3^ab^	25.4 ± 1.0^a^	9.80 ± 0.46	74.6 ± 2.1^bc^	85.1 ± 2.1^b^	92.7 ± 1.8^ab^
Pr >F^*^						
ANOVA	<0.001	0.001	0.602	0.006	0.029	0.043
Linear	<0.001	<0.001	0.071	0.773	0.020	0.029
Quadratic	0.020	0.001	0.946	<0.001	0.267	0.047

The experimental diets were formulated to contain vitamin E at 0, 20, 40, 60, 80, 100, 120 and 240 mg/kg (designated as VE0, VE20, VE40, VE60, VE80, VE100, VE120 and VE240, respectively).

Values are the means of quadruplicate groups and are presented as mean ± SD. Values with different superscript letters in the same column are significantly different (**P* *< 0.05).

*Significance probability associated with the *F*-statistic.

^1^Apparent digestibility coefficient of dry matter. ^2^Apparent digestibility coefficient of protein. ^3^Apparent digestibility coefficient of lipid.

### Challenge test

**Fig 4:**
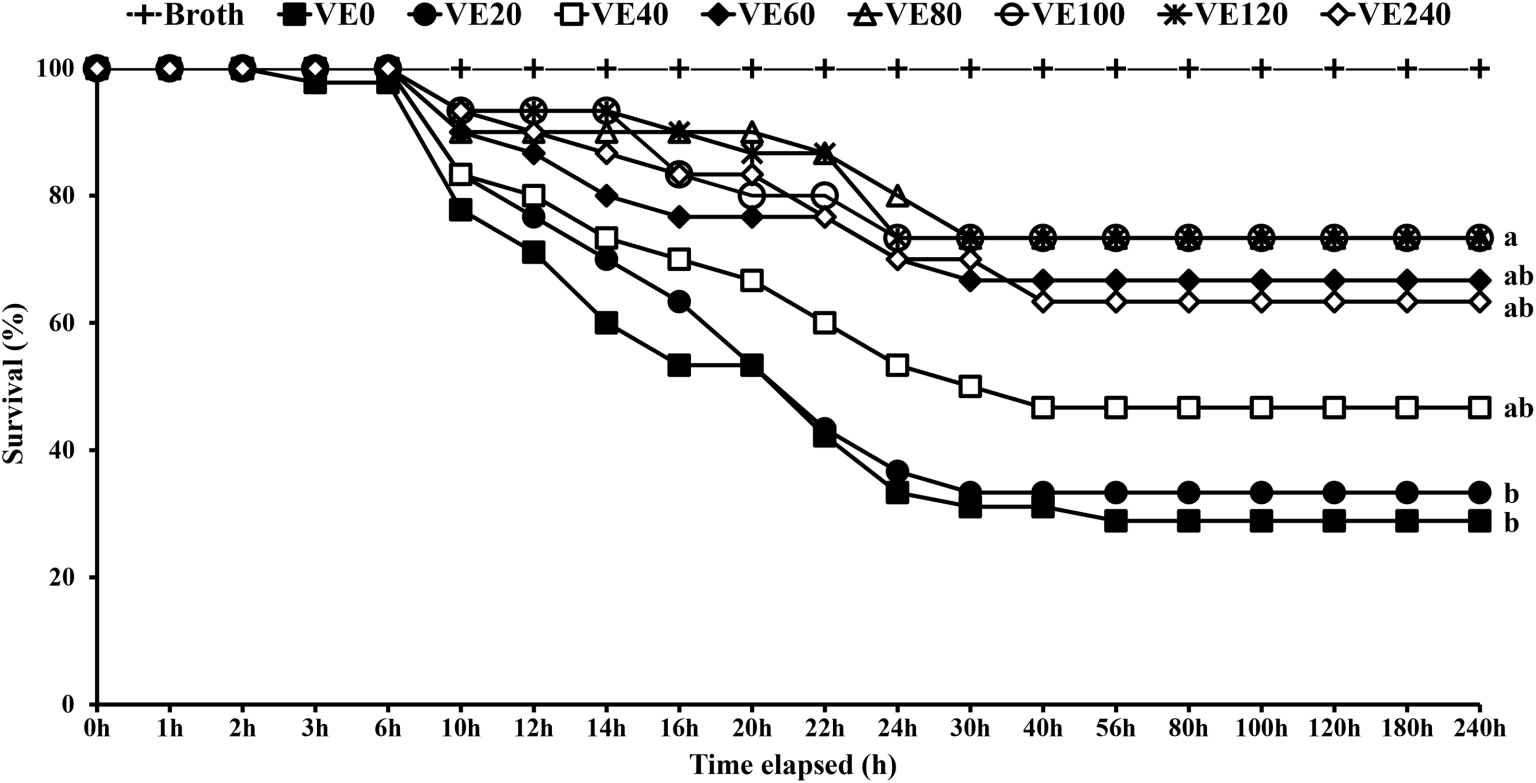
Survival of juvenile Pacific white shrimp (*Penaeus vannamei*) challenged against *Vibrio parahaemolyticus* (2.8 x 10^6^ CFU/mL) for 240 h at the end of the 56-day feeding trial. The experimental diets were formulated to contain vitamin E at 0, 20, 40, 60, 80, 100, 120 and 240 mg/kg (designated as VE0, VE20, VE40, VE60, VE80, VE100, VE120 and VE240, respectively).

After 56 days of the feeding trial, the challenge test with *V. parahaemolyticus* (2.8 × 10^6^ CFU/mL) was conducted for 240 h post-infection. From 6 h after *V. parahaemolyticus* infection, shrimp exhibited characteristic symptoms of the infection, including pallor in the abdominal region and a softened carapace. Shrimp fed VE80, VE100 and VE120 diets showed significantly higher cumulative survivals compared to shrimp fed VE0 and VE20 diets (Fig 4). The cumulative survival percentages of VE0, VE20, VE40, VE60, VE80, VE100, VE120 and VE240 groups were 28.9, 30.0, 46.7, 66.7, 73.3, 73.3, 73.3 and 63.3%, respectively. The negative control (Broth) did not show any mortality.

## Discussion

In our results, optimal dietary VE level significantly enhanced the growth and survival of the shrimp. This positive trend aligns with the findings from other studies, including oriental river prawn [11], black tiger shrimp [9] and grass carp [7]. Notably, crustacean species have shown optimal growth performance with dietary VE levels in the range of 70-100 mg/kg, e.g., 80 mg/kg for black tiger shrimp [9], 99 mg/kg for Pacific white shrimp [14] and 80 mg/kg for oriental river prawn [11]. As a lipid-soluble antioxidant, VE plays an important role in maintaining hepatopancreas health. Li et al. [11] demonstrated that dietary supplementation of optimal VE level promoted the development of ultrastructure of the hepatopancreas of B cells and R cells by increasing the abundance of endoplasmic reticulum, Golgi bodies and mitochondria in hepatocytes of oriental river prawn. Consistent with our results, Hamre et al. [26] observed a reduction in hepatic MDA levels with increased dietary VE supplementation. Hamre [27] reported that acute VE deficiency in fish manifests as the accumulation of yellow-colored autofluorescent material (ceroids) in hepatocyte vacuoles, liver discoloration and hepatocyte necrosis. Similarly, the darkening of the hepatopancreas is a recognized sign of VE deficiency in Pacific white shrimp [28]. The effect of VE on hepatopancreas health might have beneficially affected growth of the shrimp since the hepatopancreas is the main organ and is responsible for nutrient metabolism and storage in the shrimp.

Moderate dietary VE supplementation enhanced the growth performance of the shrimp in the present study. In contrast, both lower or excessively high VE inclusion levels negatively impacted the growth performance. The finding is consistent with previous reports indicating that high doses of dietary α-tocopherol acetate can impair the growth in fishes such as Caspian trout (*Salmo caspius*) [29], grass carp [7] and northern whiting (*Sillago sihama*) [30]. VE toxicity could lead to bleeding, intracranial hemorrhage, neurologic diseases and anemia, adversely affecting growth, immune functions, feed efficiency and nutrient interactions [31]. Fang et al. [32] reported that VE is stored in lipid-rich organs. Excessive dietary VE (10,000 mg/kg) has been shown to promote lipid peroxidation while increasing blood hydroperoxide accumulation and reducing erythrocyte osmotic fragility in sweet smelt (*Plecoglossus altivelis*) [33]. In the present study, hemolymph and hepatopancreas VE levels were increased linearly with a gradual increase in dietary VE levels. This observation may be due to VE being a fat-soluble vitamin, which is slowly eliminated from the body through excretion [34].

In the present study, moderate dietary VE supplementation significantly elevated non-specific immune parameters and the expression of antioxidant enzyme-related genes in the shrimp. NBT activity is a measure of phagocytotic activity, which produces highly reactive ROS in immune cells to combat pathogenic microbes in teleosts. Lysozyme, a lytic enzyme, plays a crucial role in destroying bacterial cell walls by lysing peptidoglycan and activating autolytic enzymes in bacteria or parasites [35]. Antiprotease activity is essential for inhibiting proteases secreted by bacteria during bacterial infections [36]. In our results, dietary VE level at 60-83 mg/kg significantly increased the activities of immune parameters in the shrimp compared to both higher and lower inclusion levels. Similar findings were reported for turbot [37] and oriental river prawn [11]. Although the exact mechanism remains unclear, Hamre [27] suggested that VE may improve phagocyte populations and immunostimulation by maintaining membrane fluidity. Additionally, VE could protect membrane polyunsaturated fatty acids from peroxidation [2]. PO catalyzes the melanin production process in invertebrates and is involved in the opsonization of pathogens for phagocytic uptake [38]. Hu et al. [39] reported that VE supplementation could maintain high serum PO activity in Pacific white shrimp. VE is known to upregulate prophenol-activating enzymes, precursors of PO [40], which may have contributed to the increased PO activity observed in the present study. MDA level is a marker of oxidative stress in tissues [41]. In the present study, VE80 group showed a significantly lower hepatopancreatic MDA level than other groups, indicating that moderate dietary VE is effective in reducing tissue oxidative damage. Ding et al. [42] and Chen et al. [43] observed a similar trend in cobia (*Rachycentron canadum*) and Chinese mitten crab fed diets with varying VE levels. VE is a well-established chain-breaking antioxidant that scavenges ROS, which likely explains the reduced MDA levels observed with increased dietary VE intake [44]. However, levels exceeding 120 mg/kg of VE significantly elevated MDA levels. Future studies are recommended to elucidate the underlying mechanisms.

SOD converts superoxide or singlet oxygen radicals into hydrogen peroxide or oxygen molecules, whereas GPx reduces hydrogen peroxide and lipid peroxides to water or corresponding alcohols [45]. In particular, the antioxidant enzyme *C-MnSOD* plays a crucial role in protecting mitochondria from oxidative damage. Sun et al. [46] reported that elevated dietary lipid and VE levels upregulated the gene expression of *SOD* and *GPx*. They suggested that antioxidant enzyme synthesis-related gene expressions might have increased as a response to the increased lipid peroxide levels, lipophagy and apoptosis in the hepatopancreas. Pan et al. [7] demonstrated that dietary VE supplementation significantly enhanced the antioxidant activity in the spleen, skin and head kidney of grass carp. Additionally, vitamins A, C, and E were highly effective in reducing pesticide-induced hepatotoxicity in rats by improving the antioxidant enzyme activity and scavenging free radicals [47]. In line with these findings, this study observed upregulated gene expressions of *C-MnSOD* and *GPx*. Previous studies have demonstrated that excessive dietary VE levels can lead to numerous complications in fish and shrimp species, including increased hepatic MDA levels, inhibition of blood clotting, impaired immune function, induction of cytochrome P450, accelerated metabolism of other chemicals and increased mortality [48–50]. These adverse effects may have contributed to the observed retarded growth, impaired immune activities, elevated MDA levels and reduced disease resistance in this study.

In the present study, ADC values were positively correlated with the growth performance. ADC of each nutrient represents the proportion absorbed from the diet and its efficiency in nutrient utilization. Gao et al. [51] found that feed digestibility decreases when the diet contains higher levels of oxidized lipids indicating that VE enhances diet digestibility and lipid uptake by preventing lipid peroxidation. Dietary VE supplementation also significantly improved intestinal structure by increasing villus height and width. The intestinal epithelium is frequently exposed to oxidative stress and inflammation due to various factors. Sivaramakrishnan et al. [52] reported that dietary VE supplementation significantly increased villus height and lymphoid cell concentration in the lamina propria of milkfish (*Chanos chanos*). Shin et al. [53] found positive correlations between villus height and expression of anti-inflammation genes, including transforming growth factor-β1 and interleukin-10. In contrast, a negative correlation was observed between villus height and the expression of pro-inflammation genes, including interleukin-8 and tumor necrosis factor-α. He et al. [54] reported that dietary VE supplementation significantly increased intestinal somatostatin levels in channel catfish (*Ictalurus punctatus*), which helps protect intestinal epithelial cells from necrosis and damage caused by harmful substances. Additionally, Ahmed et al. [55] observed that feeding an optimal VE level diet increased the villus height and width, thereby enhancing interleukin-10 expression in Nile tilapia (*Oreochromis niloticus*). Accordingly, dietary VE supplementation may have enhanced the villus structure of shrimp in our findings by maintaining the integrity and functionality of cell membranes, serving as an effective lipid-soluble antioxidant.

AHPND, caused by toxins encoded by the *Pir*A- and *Pir*B-like genes in *V. parahaemolyticus* results in mass mortalities in shrimp culture [56]. In the present study, VE was found to enhance disease tolerance in Pacific white shrimp against *V. parahaemolyticus*, a finding consistent with previous studies showing that dietary VE improves pathogen resistance in various fishes, including grass carp [7], Nile tilapia [57], rainbow trout (*Oncorhynchus mykiss*) [58] and milkfish [59]. *V. parahaemolyticus* primarily affects the hepatopancreas of shrimp. VE exhibits numerous hepatoprotective effects, such as antioxidative potential, prevention of lipid peroxidation, enhancement of antioxidative enzyme activity and support for cell membrane development [26]. An improved survival was observed in VE80, VE100 and VE120 groups after the challenge test. The VE inclusion level also significantly enhanced immunity and upregulated the gene expression of *Crustin*, *C-MnSOD* and *GPx*. Consistent with our findings, it has been reported that an increase in non-specific immunity and antioxidant capacity in Pacific white shrimp enhances survival against *V. parahaemolyticus* infections [60]. The findings suggest that an optimal level of dietary VE could enhance non-specific immunity and antioxidant capacity, thereby improving the survival of juvenile shrimp challenged with *V. parahaemolyticus*.

While the present study provides valuable insights, it was conducted under controlled laboratory conditions, which may not reflect the practical environmental conditions of commercial shrimp farming systems. Additionally, further research is needed to ascertain whether the observed effects are consistent across different life stages of Pacific white shrimp.

## Conclusion

Overall, the results indicate that the inclusion of an optimum level of VE in diets enhanced growth performance, non-specific immunity, antioxidant status, intestinal morphology and nutrient digestibility in juvenile Pacific white shrimp. Based on growth performance, the optimal dietary VE requirement is approximately 72 mg/kg. In addition, the disease resistance results from the *V. parahaemolyticus* challenge test suggest that an optimal dietary VE level may help reduce the incidence of EMS caused by AHPND.
